# Clinical Evaluation of a Rapid Reciprocal-Flow PCR Assay and Real-Time PCR Assay with Quenching Probe for Detection of *Mycobacterium tuberculosis* Complex

**DOI:** 10.3390/microorganisms13010201

**Published:** 2025-01-17

**Authors:** Kosuke Kosai, Keisuke Matsumoto, Takahisa Ishikawa, Yasuhide Kawamoto, Norihiko Akamatsu, Kenji Ota, Fujiko Mitsumoto-Kaseida, Norihito Kaku, Hiroo Hasegawa, Koichi Izumikawa, Hiroshi Mukae, Katsunori Yanagihara

**Affiliations:** 1 Department of Laboratory Medicine, Nagasaki University Graduate School of Biomedical Sciences, 1-7-1 Sakamoto, Nagasaki 852-8501, Japan; kaseida@nagasaki-u.ac.jp (F.M.-K.);; 2 Department of Laboratory Medicine, Nagasaki University Hospital, Nagasaki 852-8501, Japanhhase@nagasaki-u.ac.jp (H.H.); 3 Department of Infectious Diseases, Nagasaki University Graduate School of Biomedical Sciences, Nagasaki 852-8501, Japan; 4 Department of Respiratory Medicine, Nagasaki University Graduate School of Biomedical Sciences, Nagasaki 852-8501, Japan; hmukae@nagasaki-u.ac.jp

**Keywords:** molecular diagnosis, rapid detection, reciprocal-flow PCR, quenching probe

## Abstract

This study investigated the diagnostic efficiencies of two assays for the detection of *Mycobacterium tuberculosis* complex: (1) the reciprocal-flow real-time polymerase chain reaction (PCR)-based GeneSoC assay and (2) the real-time PCR based GENECUBE MTB assay with quenching probe. These assays were performed for stored clinical samples and results were compared with the confirmed results based on culture and COBAS TaqMan MTB assay. A total of 53 samples (20 confirmed positives and 33 confirmed negatives) were included in the performance analysis. The GeneSoC assay showed concordance in all 53 samples, regardless of specimen type, while the GENECUBE MTB assay showed concordance in 19 of the 20 confirmed positive samples and all 33 confirmed negative samples. The overall agreement was 100.0% for the GeneSoC assay and 98.1% for the GENECUBE MTB assay. Positive and negative percent agreements were 100.0% each for the GeneSoC assay and 95.0% and 100.0%, respectively, for the GENECUBE MTB assay. Both the GeneSoC and GENECUBE MTB assays exhibited excellent performance in detecting *M. tuberculosis* complex. The GeneSoC assay is useful for independent assays of individual samples, whereas the GENECUBE MTB assay is suitable for batch assays of multiple samples.

## 1. Introduction

Tuberculosis, caused by *Mycobacterium tuberculosis*, is a significant public health concern worldwide. In addition to culture, which is the standard method for tuberculosis diagnosis, several molecular methods, including polymerase chain reaction (PCR), have been developed for the diagnosis of tuberculosis. These molecular methods have enabled rapid and sensitive detection of *M. tuberculosis* and are part of the diagnostic algorithm [1,2].

The COBAS TaqMan MTB assay, a widely used molecular test for *M. tuberculosis* detection [3], is a real-time PCR assay that amplifies part of the 16S rRNA gene using a TaqMan probe [4]. Compared with culture, the COBAS TaqMan MTB test exhibits sensitivity and specificity rates of 79.1–82.4% and 97.7–98.2%, respectively [1,4], and 60.0–91.5% and 95.8–100.0%, respectively, especially for respiratory specimens [1,3,4,5,6,7].

The GENECUBE MTB assay detects *M. tuberculosis* complex within 60 min using a fully automated genetic analyzer (GENECUBE system). This assay is based on real-time PCR and uses quenching probe (QProbe) to detect *M. tuberculosis* complex DNA. The GENECUBE MTB assay amplifies *dnaJ*, which is found in all mycobacteria, and confirms the presence of *M. tuberculosis* complex using Qprobes [8,9]. This assay can examine 24 samples and four items simultaneously in a short time. Compared with culture, the sensitivity and specificity of the assay in detecting the *M. tuberculosis* complex are 86.2% and 96.6%, respectively, for respiratory specimens and 85.4% and 99.8%, respectively, for respiratory and non-respiratory specimens [8]. In addition to *M. tuberculosis* complex, the GENECUBE system has been applied for the detection of other pathogens and toxins, such as *M. avium*, *M. intracellulare*, *Mycoplasma pneumoniae*, *Staphylococcus aureus* and *mecA*, *tcdB* in *Clostridioides difficile*, severe acute respiratory syndrome coronavirus 2 (SARS-CoV-2), and influenza virus [10,11,12,13,14].

GeneSoC is a rapid reciprocal-flow PCR system that facilitates gene amplification within 15 min using microfluidic thermal cycling. In the GeneSoC system, a small amount of PCR solution is reciprocated across zones at different temperatures for thermal cycling, enabling rapid gene amplification [15]. This assay can be performed independently for individual samples. This system has been used to detect *S. aureus* and methicillin resistance, SARS-CoV-2, and the influenza virus [16,17,18,19,20]. Recently, the GeneSoC system has been applied for the detection of the *M. tuberculosis* complex. This GeneSoC assay targets insertion sequence (IS) 6110 which is exclusively observed in *M. tuberculosis* complex and is an important marker for differentiating it from other mycobacteria [9].

In this study, we aimed to evaluate the performance of GeneSoC and GENECUBE MTB assays in detecting *M. tuberculosis* complex in clinical specimens at a Japanese tertiary hospital.

## 2. Materials and Methods

### 2.1. Samples and Routine Testing

We randomly chose samples with sufficient volume among non-consecutive samples, which were submitted for routine testing between 2011 and 2022 at the Nagasaki University Hospital for the detection of *M tuberculosis* and stored at −30 °C. We included the multiple samples obtained from a single patient if specimen types were different. The clinical samples were stored after treatment with N-acetyl-l-cysteine-sodium hydroxide (NALC-NaOH). For routine testing, acid-fast staining, culture using Ogawa’s media, and real-time PCR assays using COBAS TaqMan MTB (Roche Diagnostics, Rotkreuz, Switzerland) were performed for all samples. Liquid culture using a Mycobacterial Growth Indicator Tube (BD, Sparks, MD, USA) on a BD BACTEC MGIT 960 automated mycobacterial detection system (BD, Sparks, MD, USA) was performed for samples with positive results for acid-fast staining or COBAS TaqMan MTB assay. Routine test results were obtained from a clinical laboratory database.

### 2.2. Retest Using the COBAS TaqMan MTB Assay

Retest using the COBAS TaqMan MTB assay was performed for stored clinical samples to confirm the results of routine testing. For DNA extraction, 100 µL of the stored clinical samples were mixed with 500 µL of wash buffer and centrifuged at 12,500× *g* for 10 min. The pellet was mixed with 100 µL of bacterial cell lysis buffer, and the suspension was incubated at 60 °C for 10 min. After mixing with 100 µL of neutralization buffer and centrifuging at 10,500× *g* for 1 min, supernatant was used as template for amplification. Then, COBAS TaqMan MTB assay was performed using a total reaction volume of 100 µL containing 50 µL each of template and prepared PCR master mix on the COBAS TaqMan 48 system.

### 2.3. Confirmed Results

Samples with confirmed positive or negative results were defined as those showing fully positive or negative concordance among the three methods: culture for routine testing, COBAS TaqMan MTB assay for routine testing, and COBAS TaqMan MTB assay for retesting.

### 2.4. DNA Extraction for GeneSoC and GENECUBE MTB Assays

First, 400 µL of the stored clinical samples were treated by bead-beating at 3000 rpm for 3 min and incubated at 95 °C for 10 min. DNA was extracted from the suspension using MagDEA DX SV and magLEAD 12gC (Precision System Science Co., Ltd., Chiba, Japan), a fully automated nucleic acid extraction system, and eluted with 50 µL of elution buffer for GENECUBE MTB assay or sterile distilled water for GeneSoc assay.

### 2.5. GeneSoC Assay

GeneSoC system is a multiplex real-time PCR system that detects fluorescence intensity during reactions. PCR solutions are introduced into a microchannel on a chip and reciprocally flow between multiple preheated zones, enabling rapid temperature changes in the solutions in a short time [15,16]. The GeneSoC assay was performed using 18 of 20 µL volume mixture containing 5 µL of template, 3 µL of primers and probes, and 12 µL of prepared PCR master mix under the following conditions: 5 s at 94 °C, 50 cycles of 6 s at 94 °C, and 8 s at 61 °C.

### 2.6. GENECUBE MTB Assay

Amplification and melting curve analyses were performed automatically using the GENECUBE system. Briefly, *dnaJ* was amplified using 4 µL of template, 5.2 µL of primers and probes, and 4 µL of KOD mix under the following conditions: 30 s at 94 °C, 50 cycles of 1 s at 97 °C, 3 s at 58 °C, and 5 s at 63 °C. The melting curve was subsequently analyzed using Qprobe under the following conditions: 30 s at 94 °C, 30 s at 39 °C, and heating up from 40 °C to 75 °C at a rate of 0.09 °C/s [8].

### 2.7. Performance of the GeneSoC and GENECUBE MTB Assays and Statistical Analysis

Results of the GeneSoC and GENECUBE MTB assays were compared with the confirmed results. Positive and negative percent agreements (PPA and NPA, respectively) and overall agreement (OA) were calculated as follows: PPA = number of concordant positive results divided by the number of all confirmed positive results, NPA = number of concordant negative results divided by the number of all confirmed negative results [21,22], and OA = number of concordant positive and negative results divided by the number of confirmed positive and negative results. Additionally, 95% confidence interval (CI) was calculated using the R version 4.4.2 software [23]. Agreement or difference in diagnostic performance between the methods was evaluated by Kappa statistics or McNemar’s test using JMP v16 (SAS Institute Inc., Cary, NC, USA) [24].

## 3. Results

### 3.1. Determination of Confirmed Results and Exclusion of a Sample with an Invalid Result

To determine the samples with confirmed results, stored clinical samples were retested using the COBAS TaqMan MTB assay and the results were compared with those of the culture and COBAS TaqMan MTB assay performed as routine testing. Of the 64 samples examined, 21 and 33 were concordantly positive and negative, respectively, among the three results and defined as confirmed positive and negative samples, respectively. Meanwhile, 10 samples were considered as non-confirmed samples. Of the 54 samples with confirmed results, one sample that tested positive using the GeneSoC assay but showed an invalid result using the GENECUBE MTB assay was excluded from the analysis. Table 1 shows the concordance among the three methods: culture for routine testing, COBAS TaqMan MTB assay for routine testing, and COBAS TaqMan MTB assay for retesting, for the 63 samples finally included. A total of 53 samples (20 confirmed positives and 33 confirmed negatives), consisting of 31 respiratory and 22 non-respiratory specimens (seven pleural fluids, three pus samples, three cerebrospinal fluids, two gastric fluids, one stool sample, one synovial fluid, one pericardial fluid, and four others), were considered as samples with confirmed results, whereas 10 samples, consisting of five respiratory and five non-respiratory specimens (one pleural fluid, one pus sample, and three stool samples), were considered as samples with non-confirmed results.

### 3.2. GeneSoC and GENECUBE MTB Assays for Samples with Confirmed Results

Table 2 and Table 3 show the concordance of the results for the detection of *M. tuberculosis* complex. Full positive and negative concordance among GeneSoC assay, GENECUBE MTB assay, and confirmed results were observed in 19 and 33 samples, respectively. Of the confirmed positive samples that tested positive using the GeneSoC assay, one respiratory sample tested negative in the GENECUBE MTB assay (Table 2). In the GeneSoC assay, concordance was observed for all 53 samples (20 confirmed positives and 33 confirmed negatives) regardless of the specimen types (12 confirmed positives and 19 confirmed negatives for respiratory specimens; 8 confirmed positives and 14 confirmed negatives for non-respiratory specimens). In the GENECUBE MTB assay, concordance was observed for 19 of the 20 confirmed positive samples and all 33 confirmed negative samples. One false-negative result was observed in the GENECUBE MTB assay for a respiratory specimen (Table 3).

### 3.3. Performance of the GeneSoC and GENECUBE MTB Assays

Table 4 shows the agreements between the GeneSoC or GENECUBE MTB assay results and the confirmed results. For all specimens, the OA was 100.0% (53/53; kappa, 1.00; 95% CI, 1.00–1.00) between the GeneSoC assay results and the confirmed results, and the PPA and NPA between them were 100.0% each, whereas the OA was 98.1% (52/53; kappa, 0.96; 95% CI, 0.88–1.00) between the GENECUBE MTB assay results and the confirmed results, and the PPA and NPA between them were 95.0% and 100.0%, respectively. Between the GeneSoC and GENECUBE MTB assays, the OA for all specimens was 98.1% (52/53; kappa, 0.96; 95% CI, 0.88–1.00), and the PPA and NPA for them were 95.0% and 100.0%, respectively.

The analysis of diagnostic performance for all specimens using McNemar’s test showed no significant difference between the GeneSoC assay results and the confirmed results (*p* = 1.0000), between the GENECUBE MTB assay results and the confirmed results (*p* = 0.3173), and between the GeneSoC and GENECUBE MTB assay results (*p* = 0.3173).

### 3.4. GeneSoC and GENECUBE MTB Assays for Samples with Non-Confirmed Results

The GeneSoC and GENECUBE MTB assays were performed for the remaining 10 samples that showed discordance among culture for routine testing, COBAS TaqMan MTB assay for routine testing, and COBAS TaqMan MTB assay for retesting (Table 1). Table 5 shows the detection results using the GeneSoC and GENECUBE assays for 10 samples with non-confirmed results. Among the five samples that tested positive in routine culture and COBAS TaqMan MTB assay but negative in the retest using the COBAS TaqMan MTB assay, four tested positive in the GeneSoC assay but none tested positive in the GENECUBE MTB assay. In addition, two samples, which tested positive in routine testing and retest using the COBAS TaqMan MTB assay but negative in routine culture, tested positive in both GeneSoC and GENECUBE MTB assays. Of the three samples that tested positive in routine testing using the COBAS TaqMan MTB assay but negative in routine culture and retest using the COBAS TaqMan MTB assay, all three tested positive in the GeneSoC assay and two tested positive in the GENECUBE MTB assay.

## 4. Discussion

This study showed excellent concordance between the GeneSoC or GENECUBE MTB assay results and the confirmed results, with an OA of 100.0% (kappa, 1.00) or 98.1% (kappa, 0.96) for all specimens. No significant difference was observed between the GeneSoC assay results, the GENECUBE MTB assay results, and the confirmed results (*p* = 0.3173–1.0000 using McNemar’s test). In particular, the PPAs of these assays in this study (GeneSoC, 100.0%; GENECUBE MTB, 95.0%) were higher than the sensitivities reported in previous studies for the COBAS TaqMan MTB and GENECUBE MTB assays compared with the culture (79.1–82.4% and 85.4%, respectively) [1,4,8]. A previous study reported that the sensitivity for respiratory specimens (88.4%) was higher than that for non-respiratory specimens (63.6%) when comparing the COBAS TaqMan MTB assay with the culture [4]. However, in this study, GeneSoC and GENECUBE MTB assays exhibited high PPAs compared with the confirmed results, regardless of the specimen type (Table 4). To ensure the accuracy of the confirmed results, we defined samples with full concordance among the three methods (culture, COBAS TaqMan MTB assay for routine testing, and COBAS TaqMan MTB assay for retest) as samples with confirmed results in this study. High PPAs in this study may be due to the differences in the comparator methods used in this study, compared with previous studies.

We observed one confirmed positive sample that tested positive using the GeneSoC assay but showed an invalid result using the GENECUBE MTB assay, and we excluded it from the analysis. Meanwhile, a previous study showed that some samples showed valid results using the GENECUBE MTB assay but were determined to be invalid using the COBAS AMPLICOR assay. These invalid results might be due to the inhibition of amplification [8].

We used stored clinical samples for this study and observed non-confirmed results for 10 samples (Table 1). Of these 10 non-confirmed samples, eight, regardless of routine culture results, tested positive in routine testing but negative in retest using the COBAS TaqMan MTB assay. Storage conditions might have influenced the difference in results between routine testing and retest using the COBAS TaqMan MTB assay. Of these eight samples, seven tested positive using the GeneSoC assay and two tested positive using the GENECUBE MTB assay (Table 5). In addition, one confirmed positive sample tested positive using the GeneSoC assay but negative using the GENECUBE MTB assay (one false-negative result in the GENECUBE MTB assay) (Table 2 and Table 3). These differences in detection results between the GeneSoC and GENECUBE MTB assays might be due to the difference in assay targets (IS6110 for the GeneSoC assay and *dnaJ* for the GENECUBE MTB assay) or in assay principles.

This study has some limitations. First, this study included a small sample size. Further studies using larger sample sizes are required to enhance our results. Second, we used stored samples and storage conditions might have affected the assay results. Use of fresh samples might increase the accuracy of assay results.

Overall, the GeneSoC assay exhibited excellent performance in detecting the *M. tuberculosis* complex. This assay can be used for individual patients in the clinical setting as it can be performed rapidly, easily, and independently for each sample. The GENECUBE MTB assay also showed accuracy and can be used for batch assays of multiple samples. Both assays can be used complementarily depending on the requirements of clinical laboratories.

## Figures and Tables

**Table 1:** Concordance between the culture and COBAS TaqMan MTB assay results for the detection of *M. tuberculosis* complex.

Culture (Routine)	COBAS TaqMan MTB		No. of Specimens		
	Routine	Retest	All	Respiratory	Non-Respiratory
Positive	Positive	Positive	20	12	8
Positive	Positive	Negative	5	4	1
Negative	Positive	Positive	2	0	2
Negative	Positive	Negative	3	1	2
Negative	Negative	Negative	33	19	14

**Table 2:** Detection results of *M. tuberculosis* complex using the GeneSoC and GENECUBE assays for 53 samples with confirmed results.

Confirmed Result	GeneSoC	GENECUBE	No. of Specimens		
			All	Respiratory	Non-Respiratory
Positive	Positive	Positive	19	11	8
Positive	Positive	Negative	1	1	0
Negative	Negative	Negative	33	19	14

**Table 3:** Concordance between the GeneSoC or GENECUBE assay results and confirmed results for the detection of *M. tuberculosis* complex.

Specimen	Confirmed Result	GeneSoC		GENECUBE	
		Positive	Negative	Positive	Negative
All	Positive	20	0	19	1
	Negative	0	33	0	33
Respiratory	Positive	12	0	11	1
	Negative	0	19	0	19
Non-respiratory	Positive	8	0	8	0
	Negative	0	14	0	14

**Table 4:** Agreement between the GeneSoC or GENECUBE assay results and the confirmed results for the detection of *M. tuberculosis* complex.

Specimen	Method	OA (95% CI)	PPA (95% CI)	NPA (95% CI)
All	GeneSoC	100.0 (93.3–100.0)	100.0 (83.2–100.0)	100.0 (89.4–100.0)
	GENECUBE	98.1 (89.9–100.0)	95.0 (75.1–99.9)	100.0 (89.4–100.0)
Respiratory	GeneSoC	100.0 (88.8–100.0)	100.0 (73.5–100.0)	100.0 (82.4–100.0)
	GENECUBE	96.8 (83.3–99.9)	91.7 (61.5–99.8)	100.0 (82.4–100.0)
Non-respiratory	GeneSoC	100.0 (84.6–100.0)	100.0 (63.1–100.0)	100.0 (76.8–100.0)
	GENECUBE	100.0 (84.6–100.0)	100.0 (63.1–100.0)	100.0 (76.8–100.0)

OA, overall agreement; PPA, positive percent agreement; NPA, negative percent agreement; CI, confidence interval.

**Table 5:** Detection results of *M. tuberculosis* complex using the GeneSoC and GENECUBE assays for 10 samples with non-confirmed results.

Culture (Routine)	COBAS TaqMan MTB		All	GeneSoC		GENECUBE	
	Routine	Retest		Positive	Negative	Positive	Negative
Positive	Positive	Negative	5	4	1	0	5
Negative	Positive	Positive	2	2	0	2	0
Negative	Positive	Negative	3	3	0	2	1

## Data Availability

The original contributions presented in this study are included in the article. Further inquiries can be directed to the corresponding author.

## References

[1] Kim J.H., Kim Y.J., Ki C.S., Kim J.Y., Lee N.Y. (2011). Evaluation of Cobas TaqMan MTB PCR for detection of *Mycobacterium tuberculosis*. J. Clin. Microbiol..

[2] Procop G.W. (2016). Laboratory Diagnosis and Susceptibility Testing for *Mycobacterium tuberculosis*. Microbiol. Spectr..

[3] Park K.S., Kim J.Y., Lee J.W., Hwang Y.Y., Jeon K., Koh W.J., Ki C.S., Lee N.Y. (2013). Comparison of the Xpert MTB/RIF and Cobas TaqMan MTB assays for detection of *Mycobacterium tuberculosis* in respiratory specimens. J. Clin. Microbiol..

[4] Bloemberg G.V., Voit A., Ritter C., Deggim V., Bottger E.C. (2013). Evaluation of Cobas TaqMan MTB for direct detection of the *Mycobacterium tuberculosis* complex in comparison with Cobas Amplicor MTB. J. Clin. Microbiol..

[5] Yang Y.C., Lu P.L., Huang S.C., Jenh Y.S., Jou R., Chang T.C. (2011). Evaluation of the Cobas TaqMan MTB test for direct detection of *Mycobacterium tuberculosis* complex in respiratory specimens. J. Clin. Microbiol..

[6] Lee M.R., Chung K.P., Wang H.C., Lin C.B., Yu C.J., Lee J.J., Hsueh P.R. (2013). Evaluation of the Cobas TaqMan MTB real-time PCR assay for direct detection of *Mycobacterium tuberculosis* in respiratory specimens. J. Med. Microbiol..

[7] Park J.E., Huh H.J., Koh W.J., Song D.J., Ki C.S., Lee N.Y. (2017). Performance evaluation of the Cobas TaqMan MTB assay on respiratory specimens according to clinical application. Int. J. Infect. Dis..

[8] Hida Y., Hisada K., Shimada A., Yamashita M., Kimura H., Yoshida H., Iwasaki H., Iwano M. (2012). Rapid detection of the *Mycobacterium tuberculosis* complex by use of quenching probe PCR (geneCube). J. Clin. Microbiol..

[9] Chin K.L., Sarmiento M.E., Norazmi M.N., Acosta A. (2018). DNA markers for tuberculosis diagnosis. Tuberculosis.

[10] Yoshida S., Tsuyuguchi K., Kobayashi T., Shimatani Y., Inoue Y. (2022). Effect of sputum quality on *Mycobacterium avium-intracellulare* complex lung disease diagnosis and treatment initiation according to disease type. Diagn. Microbiol. Infect. Dis..

[11] Ito Y., Iwashima S., Hayano S., Nishio T., Shiozawa R., Yata S., Kubota T., Kubota A., Uemura K. (2018). Rapid detection of the macrolide sensitivity of pneumonia-causing *Mycoplasma pneumoniae* using quenching probe polymerase chain reaction (GENECUBE^®^). Mol. Diagn. Ther..

[12] Hida Y., Uemura K., Sugimoto H., Kawashima Y., Koyanagi N., Notake S., Akashi Y., Sakaguchi S., Kimura H., Suzuki H. (2019). Evaluation of performance of the GENECUBE assay for rapid molecular identification of *Staphylococcus aureus* and methicillin resistance in positive blood culture medium. PLoS ONE.

[13] Hara T., Suzuki H., Oyanagi T., Koyanagi N., Ushiki A., Kawabata N., Goto M., Hida Y., Yaguchi Y., Tamai K. (2020). Clinical evaluation of a non-purified direct molecular assay for the detection of *Clostridioides difficile* toxin genes in stool specimens. PLoS ONE.

[14] Kiyasu Y., Akashi Y., Sugiyama A., Takeuchi Y., Notake S., Naito A., Nakamura K., Ishikawa H., Suzuki H.A. (2021). A prospective evaluation of the analytical performance of GENECUBE^®^ HQ SARS-CoV-2 and GENECUBE^®^ FLU A/B. Mol. Diagn. Ther..

[15] Furutani S., Naruishi N., Hagihara Y., Nagai H. (2016). Development of an on-site rapid real-time polymerase chain reaction system and the characterization of suitable DNA polymerases for TaqMan probe technology. Anal. Bioanal. Chem..

[16] Chiba M., Aoyagi T., Yoshida M., Katsumi M., Fujimaki S.I., Ishii Y., Tateda K., Kaku M. (2023). Evaluation of the performance of GeneSoC^®^, a novel rapid real-time PCR system, to detect *Staphylococcus aureus* and methicillin resistance in blood cultures. J. Infect. Chemother..

[17] Sakai J., Tarumoto N., Orihara Y., Kawamura R., Kodana M., Matsuzaki N., Matsumura R., Ogane K., Kawamura T., Takeuchi S. (2020). Evaluation of a high-speed but low-throughput RT-qPCR system for detection of SARS-CoV-2. J. Hosp. Infect..

[18] Watanabe R., Asai S., Kakizoe H., Saeki H., Masukawa A., Miyazawa M., Ohtagawa K., Ravzanaaadii M.A., Doi M., Atsumi H. (2021). Evaluation of the basic assay performance of the GeneSoc^®^ rapid PCR testing system for detection of severe acute respiratory syndrome coronavirus 2. PLoS ONE.

[19] Ota K., Kurahara R., Tsukamoto C., Kawamoto Y., Akamatsu N., Sasaki D., Mitsumoto-Kaseida F., Sakamoto K., Kosai K., Hasegawa H. (2023). Performance of the GeneSoC rapid PCR system in detection of SARS-CoV-2 from saliva specimens. Microbiol. Spectr..

[20] Takata M., Nakamoto M., Kitaura T., Okada K., Tsuneki-Tokunaga A., Yamasaki A., Kageyama S., Burioka N., Chikumi H. (2023). Rapid Multiplex RT-PCR for Influenza A and B by Genesoc^®^, a Microfluidic PCR System. Yonago Acta Med..

[21] Kosai K., Akamatsu N., Ota K., Mitsumoto-Kaseida F., Sakamoto K., Hasegawa H., Izumikawa K., Mukae H., Yanagihara K. (2022). BioFire FilmArray Pneumonia Panel enhances detection of pathogens and antimicrobial resistance in lower respiratory tract specimens. Ann. Clin. Microbiol. Antimicrob..

[22] Murata M., Kosai K., Akamatsu N., Matsuyama Y., Oda M., Wakamatsu A., Izumikawa K., Mukae H., Yanagihara K. (2023). Diagnostic Performance of BD Phoenix CPO Detect panels for detection and classification of carbapenemase-producing Gram-negative bacteria. Microbiol. Spectr..

[23] R Core Team (2024). R: A Language and Environment for Statistical Computing.

[24] Chen J.H.K., Lam H.Y., Yip C.C.Y., Wong S.C.Y., Chan J.F.W., Ma E.S.K., Cheng V.C.C., Tang B.S.F., Yuen K.Y. (2016). Clinical evaluation of the new high-throughput Luminex NxTAG Respiratory Pathogen Panel assay for multiplex respiratory pathogen detection. J. Clin. Microbiol..

